# Health systems research in fragile and conflict-affected states: a research agenda-setting exercise

**DOI:** 10.1186/s12961-016-0124-1

**Published:** 2016-07-21

**Authors:** Aniek Woodward, Egbert Sondorp, Sophie Witter, Tim Martineau

**Affiliations:** London School of Hygiene & Tropical Medicine, London, United Kingdom; Royal Tropical Institute, Amsterdam, The Netherlands; Queen Margaret University, Edinburgh, United Kingdom; Liverpool School of Tropical Medicine, Liverpool, United Kingdom

**Keywords:** Health systems research, Research agenda, Priority setting, Fragile states, Conflict affected states

## Abstract

**Background:**

There is increasing interest amongst donors in investing in the health sectors of fragile and conflict-affected states, although there is limited research evidence and research funding to support this. Agreeing priority areas is therefore critical. This paper describes an 18-month process to develop a consultative research agenda and questions for health systems research, providing reflections on the process as well as its output.

**Methods:**

After a scoping review had been conducted, primary data was collected from August 2014 to September 2015. Data was collected using a mixture of methods, including an online survey (n = 61), two face-to-face group sessions (one with 11 participants; one with 17), email consultation (n = 18), a webinar (n = 65), and feedback via LinkedIn. Two steering committees of purposively selected experts guided the research process – a core steering committee (n = 10) and broad steering committee (n = 20). The process moved from developing broad topics and lists of research needs to grouping and honing them down into a smaller, prioritised agenda, with specific research questions associated to each topic.

**Results:**

An initial list of 146 topics was honed down to 25 research needs through this process, grouped thematically under transition and sustainability, resilience and fragility, gender and equity, accessibility, capacity building, actors and accountability, community, healthcare delivery, health workforce, and health financing. They were not ranked, as all health system areas are interdependent. The research agenda forms a starting point for local contextualisation and is not definitive.

**Conclusions:**

A wide range of stakeholders participated in the different stages of this exercise, which produced a useful starting point for health systems research agenda setting in fragile and conflict-affected states. The process of engagement may have been as valuable for building a community of researchers as the product. It is now important to drive forward the research agenda. Without both a higher profile and deeper focus for this area, there is a real risk that fragile and conflict-affected states will continue to fall behind in global health and development goals.

**Electronic supplementary material:**

The online version of this article (doi:10.1186/s12961-016-0124-1) contains supplementary material, which is available to authorized users.

## Background

Fragile and conflict-affected states (FCAS) lag behind in meeting international health goals [1, 2]. While progress can usually be achieved by implementation of well-known health strategies and technologies, in FCAS such strategies are difficult to implement because they often have weak health systems, with consequences highlighted by the Ebola crisis in West Africa [3]. More and better health system research – alongside increased funding and implementation of programmes that aim to build sustainable health systems – can be expected to contribute to strengthening health systems, meeting development goals, and ultimately improving health outcomes [4–6].

Health systems research in FCAS is a growing area of interest for researchers and donors [7–9]. However, this area of research remains relatively underdeveloped, which makes it important to have guidance about what research to focus on, as well as to ensure the most efficient use of research funds. To date, however, there has been no organised discussion or consensus-building on a global research agenda for health systems in FCAS. This study was conducted with the aim of filling this gap.

This agenda-setting exercise aimed to provide guidance for those interested in knowing what areas of health systems research in FCAS require particular attention for further enquiry and investment. This study was commissioned by the Thematic Working Group (TWG) on Health Systems in Fragile and Conflict-Affected States (HS-FCAS),^1^ which aims to promote health systems research in these contexts.

Definitions and classifications of fragile, conflict-affected, and post-conflict states vary in the literature and between development agencies. A commonly used definition for ‘fragility’ is that fragile states lack the will or capacity to meet the basic needs of their populations and to reduce poverty [10–12]. Many, but not all fragile states are affected by or emerging from conflict [10], but usually they have prolonged periods of relative stability, during which health system strengthening (HSS) agendas emerge. Newer definitions place more emphasis on the lack of a social contract between citizens and the state. For instance, the OECD proposed this definition in 2012: “*A fragile region or state has weak capacity to carry out basic governance functions, and lacks the ability to develop mutually constructive relations with society*” [13]. There is a great diversity of the extent and experiences of ‘fragility’ within fragile states [14], but while they are diverse, they have weak institutions in common [2]. Fragility, therefore, has a profound influence on health, healthcare delivery and health systems, and, conversely, health and the way healthcare is delivered has a potential positive or negative influence on fragility.

The paper describes and reflects on the process which was undertaken to develop a consultative research agenda. It also presents the results on priority research needs achieved by this study.

## Methods

### Scoping review

In the first instance, to provide background analysis for the consultation process, a scoping review was conducted (August to September 2014) with the aim of collating available published sources that identify research needs or priorities on health systems in FCAS. A detailed search strategy and flow-diagram can be found in Additional file 1. A systematic search of selected OVIDSP databases (Global Health, Medline and Embase) and hand-searches of selected journals and organisations or websites (Additional file 1) revealed that a global consensus-based research agenda setting exercise on this topic had not been conducted so far.

The review found nine studies that were sufficiently relevant for inclusion. Two identify research needs for humanitarian emergencies [15, 16], while the others discuss health system research needs in post-conflict fragile states [17–23].

Those on humanitarian emergencies did not specifically focus on health systems research but reported some health systems research needs such as health system resilience [15] and the transition from humanitarian to recovery [16]. Other included studies did clearly concentrate on health systems research needs, with two focusing on specific building blocks, namely the health workforce [20] and health financing [19]. All except one used a literature review as the main method to identify research needs, with one including conference consultations [18] and another key informant perspectives [23] in their review. The one exception [22], in their own words, “*reflects the views of a limited number of experts in the field*” (p. 9) without further specifying who these experts were.

While a literature review is a helpful tool to identify research gaps, it is, in our opinion, insufficient for setting a ‘global consensus-based’ research agenda. Moreover, when the research gap is so wide, as is the case in this area of research, it seems more crucial to answer the question ‘What are the research needs?’ rather than ‘What are the research gaps?’ Therefore, this study set out to consult a variety of stakeholders (not just academics but also local implementers, policymakers and donors) from different geographical areas (different continents and countries, including FCAS) in order to move towards a global research agenda.

### Consultative study design

This study adopted a qualitative descriptive approach using different stages and methods of data collection. Primary data collection started in August 2014 with a pilot survey and ended in September 2015 with an expert workshop.

Data was collected using a mixture of methods: an online survey, two face-to-face group sessions, online group sessions, a webinar, and feedback via the HS-FCAS LinkedIn group.^2^ An overview of each method used, including its purpose, approach and timeline, are found in Table 1.

**Table 1:** Summary of methods

Stage	Purpose	Approach	Time-line
1. Development of steering committee and agreement on methodological approach	To guide methodological development	Members from the core steering committee discussed methodological development during two workshops in London	Full day on June 4 and July 17, 2014
2. Consultation on research needs a) Online survey b) Group session at the Health Systems Global Symposium	To identify health system research needs among a global sample	A purposefully selected sample of global and national stakeholders was invited to complete the survey Panellists and attendees of the Symposium session were invited to discuss health system research needs within the group	15-minute survey was open for 2 weeks in October 2014 45-minute session was held on September 30, 2014
3. Refining and short-listing research needs	To refine and short-list identified research needs	Anonymised survey results were discussed in terms of relevance and importance among the steering committee and members of ReBUILD Consortium in online group discussions, using a Delphi technique	Discussions were open for 4 weeks in December 2014
4. Reaching consensus on research agenda	To present, discuss and create consensus on the research agenda	All participants and relevant stakeholders were invited to participate in a webinar during which survey results and an initial short-list of research needs were discussed Those not able to make the webinar were asked to provide feedback via the Health Systems in Fragile and Conflict-Affected States LinkedIn group	1-hour Webinar on May 27, 2015 LinkedIn feedback open from May to August 2015
5. Developing more specific research questions	To finalise the research agenda	Purposefully invited participants were asked to critically appraise study results and develop research questions based on identified research needs at an ‘expert workshop’ in London	2-hour discussion on September 2, 2015

**Fig. 1:**
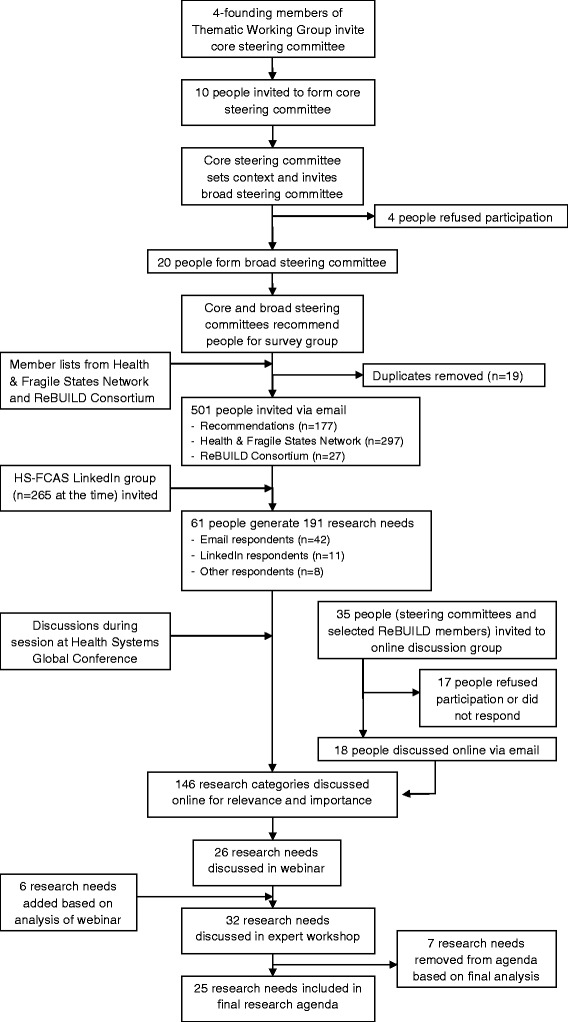
Flowchart of research agenda-setting exercise

Figure 1 shows a flowchart of this exercise including its participants at each stage. At each stage, informed consent was obtained and this study received ethical approval by the Liverpool School of Tropical Medicine (14.034). Each stage is described below.

#### Development of steering committee and agreement on methodological approach

A steering committee was set up specifically to guide the research process. This committee was divided into a ‘core’ and ‘broad’ group with the core group being those able to dedicate more time. The founding members of the TWG-HS-FCAS (n = 4) purposively selected six other members and together formed the core steering committee (n = 10), which again selected the broad steering committee (n = 20). The ‘core’ committee, which consisted mostly of academics in the United Kingdom, provided advice on methodology and contextual focus of the needs setting exercise, while the ‘broad’ committee, which consisted of a more mixed and global group, was involved in the pilot survey. Both committees were involved in some way in all of the following stages of the research.

This exercise used similar techniques as some research priority setting exercises [24–26] that identified research topics in their fields leading to a consensus-based research agenda. Two workshops in London (held on June 4 and July 17, 2014) by members of the core steering committee guided methodological development of this study. During these workshops (and feedback via email from those not able to attend) the committee decided that, because research in this area is still very underdeveloped, identification of broader research needs was going to be the focus of this exercise, while more detailed prioritisation (a stage after the identification of research needs that is often used in priority setting exercises) is more useful in the future, when the field of health systems research in FCAS is better established.

#### Consultation on research needs

Two methods were used to consult on health systems research needs and are described here separately.

##### Online survey

An online survey was used as the main method to consult on health system research needs in FCAS. The reason for using an online survey was that we could reach a global audience in a relatively short period of time. Online surveys have previously been used to identify research priorities in humanitarian emergencies [16, 24].

A pilot survey was conducted amongst the broad steering committee, which led to slight modifications. For the final survey, all contactable people with self-identified expertise in health systems in FCAS were eligible to participate. The aim was to get a sample of about 100, including a mixture of male and female participants, different types of stakeholders (donors, policymakers, academics, international and local implementers), and geographical areas (people from different continents, countries, including those from FCAS).

The survey was developed and distributed via Bristol Online Surveys. Recommended candidates by the steering committee (n = 177) together with readily available contacts of the Health & Fragile States Network^3^ (n = 297) and the ReBUILD Consortium^4^ (n = 27) were approached via email to participate in the survey. An invitation with a link to the survey was also posted on the TWG HS-FCAS LinkedIn group (which at the time had 264 members, although there was a large overlap with those emailed). The survey was open for 2 weeks (October 14–28, 2014) to allow participants to complete it at a convenient time and place. The survey was in English and took about 10–15 minutes to complete. Two reminders were sent throughout this period to encourage participation.

The survey consisted of four sections (1. Experience in HS-FCAS and research challenges; 2. Research needs; 3. Personal information; 4. Comments), with details available in Additional file 2. In total, 61 people completed the survey. Most (69 %) heard about the survey via an email invitation by the research team (42/501; 8.4 % response rate), 18 % via the HS-FCAS LinkedIn group (11/265; 4.2 % response rate), and 13 % via another channel such as a colleague. Slightly more women (59 %) than men (41 %) responded. Further, 43 % worked in international implementation (e.g. international NGOs), 31 % in academia (e.g. universities, research institutes), 16 % in local implementation (e.g. government, local NGO), and 10 % in funding (e.g. donors).

**Fig. 2:**
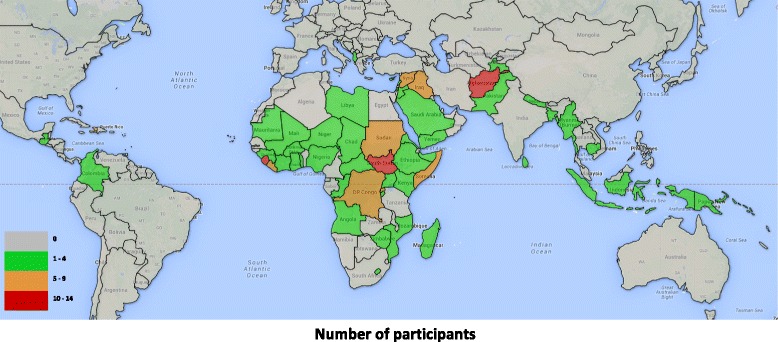
Countries where survey participants had professional experience. Number of participants

At the time of the survey, participants were living in 28 countries, of which just over half (n = 15) self-reported to be in FCAS. Most lived in the United Kingdom (12.1 %), followed by Afghanistan (8.6 %), Sierra Leone (8.6 %), and the United States of America (8.6 %). Those with experience working in FCAS (93 %) most often gained this experience in Afghanistan (8.1 %), followed by South Sudan (7 %), Sierra Leone (5.8 %), and Somalia (4.1 %). Together, participants had experience working in 56 different FCAS. Figure 2,^5^ shows a map of the world including all the countries and areas in which participants had worked. Participants were asked to list up to five countries. Those who worked in more than five countries were encouraged to list those in which they had most experience.

##### Group session at Health Systems Global Symposium

Research needs from the survey were supplemented with research needs identified during a session at the Third Global Symposium on Health Systems Research^6^ in Cape Town on September 30, 2014. The group session lasted 45 minutes and was part of a wider 2-hour session by the TWG-HS-FCAS. Panellists and attendees were invited to comment on a draft landscaping paper on health systems research in FCAS and two research papers published in the special issue of the journal *Conflict and Health*, ‘Filling the void: Health systems in fragile and conflict affected states’^7^ and to discuss health system research needs and challenges of conducting such research in FCAS.

Data from the group session comprised comments from four panellists and seven attendees. These were a mixture of men and women from different backgrounds (academic, funding, non-governmental and policy).

Results were anonymised, and the group session transcribed and analysed together with the online survey. Qualitative survey data was analysed independently by two researchers. Qualitative data was analysed thematically using deductive descriptive coding [27] with NVivo for Mac, QSR International Pty Ltd., Version 10, 2014. Quantitative data was analysed using Microsoft^®^ Excel^®^ for Mac 2011. This included analysis of sub-group differences (professional background and sex), accounted for by group size.

#### Refining and short-listing research needs

Consultation via email using a Delphi technique was the method used to refine and short-list research needs identified in the previous stage. The steering committees and selected members of the ReBUILD Consortium were approached via email for participation.

Participants (n = 18) were split in two equally sized groups in order to make the discussions more manageable and not to overload them with emails (they were required to ‘reply all’). Groups were as evenly as possible distributed in terms of sex and background (ReBUILD members were mixed with steering committee members). Most worked at universities or research institutes and therefore had a research background.

Discussions took place in three stages. In each stage, participants were asked to answer and discuss different questions, and after each stage a brief summary was provided of results from the previous stage. Participants were given about a week to respond to questions for each stage. The deadline for stage 2 was extended because of insufficient initial response. The response rate was 10 participants for each stage, with three not responding to any stage and others responding to two or all three stages. The entire process lasted 4 weeks from December 2, 2014, to January 8, 2015.

The aim of the first stage was to refine the research needs identified via the online survey and symposium and to ensure no key research needs were missing. Participants were supplied with the list of research needs (n = 191) and were asked ‘Do these results surprise you or not? Why? Do you feel any key topics are missing?’ Based on these results, some research needs were regrouped (needs were presented in categories and sub-categories) and others were added. After analysis, a list of 146 research needs was used for the following stages. The aims of these were to short-list research needs based on contextual relevance (stage 2) and importance (stage 3). Research needs found most relevant by at least two participants (n = 91) were then short-listed on importance, with 47 research needs found most important by at least two participants.

Further thematic analysis and regrouping of results from these online group sessions resulted in a list of 26 research needs across 10 themes.

#### Reaching consensus on research agenda

On May 27, 2015, the TWG-HS-FCAS organised a 1-hour webinar^8^ to present initial study findings and to invite comments and discussion in order to increase consensus on our research agenda. The webinar was advertised amongst the steering committee, survey participants who showed interest to be involved in this stage, ReBUILD Consortium members, and the HS-FCAS LinkedIn group. In total, 109 people signed up, of whom 65 attended the entire or part of the webinar.

Besides the presentations there were two 15-minute blocks of discussion open for all attendees (30 minutes in total) and on top of that a 15-minute panel discussion (in which two panellists were invited prior to the webinar to present their thoughts on our study findings). Attendees could comment or ask questions via a chat box. A technical support person compiled these and the moderator picked the most pertinent questions, which were answered by the presenters and panellists. Some of the questions that could not be answered during the webinar due to time constraints were discussed afterwards via the LinkedIn group.

All questions and discussions from the webinar and LinkedIn were used for further analysis. Based on this, six research needs and two themes were added to the agenda.

#### Developing more specific research questions

A fifth stage was added to this study, which was not in the original study design. The idea for this final stage was to transform our research needs into research questions, thereby making it more useful to potential users of this agenda. An expert workshop was organised on September 2, 2015, in London. One of the aims of this workshop was to critically appraise the results of our study and to develop research questions based on our research agenda with a group of experts. The aim was to get a mixture of stakeholders (researchers, donors, NGO workers) with expertise in health systems research in FCAS and/or setting and promoting a research agenda. Experts were purposively invited via email.

In total, 17 experts were involved in the development of research questions. Discussions took place in smaller groups to maximise individual contribution. Three participants formed an ‘online group’ which discussed via Skype. The other three groups were as evenly as possible distributed in terms of number, sex, type of stakeholder, and expertise.

The face-to-face groups were each moderated by a founding TWG member and the online group by the research assistant of this study. Each group was assigned three ‘themes’ of the research agenda and asked to transform the research needs for these themes into research questions that were specific to the context of FCAS. One person in each group was asked to take notes. Discussions lasted an hour.

Notes of all group discussions were compiled after the workshop for further analysis. Two themes were removed from the final agenda, as they were more overarching research needs, but are presented separately under ‘other research needs’ in the results section. Research questions were drawn not just from the group discussions in this final stage but also from the other research stages. These questions should be seen more as examples than final questions. Questions that most clearly reflected research needs, slightly adapted if needed, were chosen for the final agenda.

## Results and discussion

Table 2 shows the research agenda that came out of the five-staged research process. The research agenda should be seen as a starting point for further discussion. Each theme is briefly discussed here first. Although presented separately, there are linkages between most of them (for example, between equity, access and health financing). As the aim was to identify rather than prioritise research needs, those discussed first are no more important than those discussed last. After this, we reflect on the consultative process and the overall research agenda, followed by a discussion of study limitations and suggestions of ways to take this agenda forward.

**Table 2:** Research agenda on health systems in fragile and conflict-affected states (FCAS)

Themes	Research needs	Examples of research questions
Transition & sustainability	• Balance and sequence of emergency and systems strengthening • Sustainability • Reforming a post-conflict health system	• How to get the right balance between emergency service delivery and long-term systems strengthening? • How to sequence HSS in order to get enough initial stability and success to continue the long rebuilding process? • Do we need to do things differently in responding to immediate situations so that we are also supporting longer-term capacity and sustained improvements? • Is there an optimal path to sustainability of health financing after a conflict or crisis? • How to create a policy space to enable effective health system reforms after conflict?
Resilience & fragility	• Consensus on definition of ‘resilience’ • Creating resilient health systems • Relationship between health system strengthening (HSS) and fragility	• What does resilience mean in relation to health systems? How can it be measured? • How have countries survived shocks and conflicts (and if not, why not)? How can we build on these post-conflict? • What are the different types of shocks and what do these imply for coping strategies? • How to build strong local health systems? • What are the linkages with wider state-building? And what are the components and contextual factors of successful examples?
Equity & gender	• Equity issues and fragility • Relationship between more inclusive health service delivery and reduction of tension • Gender perspective and inclusion of marginalised ‘voices’	• How to integrate health equity analyses in health systems research in FCAS? Could the PROGRESS acronym (Place of residence, Race/ethnicity/culture/language, Occupation, Gender/sex, Religion, Education, Socioeconomic status, Social capital) used for analysis of disadvantaged groups in clinical trials or something similar be used or developed? • Have inclusive policies in coverage of health services contributed to lessened tensions? And if so, how? • Does targeting health programmes for women and children, and employing more women in health programmes, have any effect on lessening conflict? • How best to promote the voice of citizens in FCAS? • What methodological approaches help local people to express and exercise their views effectively?
Accessibility	• Conflict-related factors to healthcare access • Referral systems and emergency care access	• What are the key factors that influence accessibility of public services in FCAS? And to what extent are these specific to health? • What is the effectiveness of the different types of healthcare providers (public, NGO, faith-based) in these transition contexts? And how can these parallel providers best be resourced so that they contribute to the building up of a public health system? • How to improve referral systems and emergency care access to health facilities in places with limited road accessibility and non-functioning ambulance systems?
Capacity building	• Health system capacity building, particularly health workforce and leadership • Capacity building of local researchers and information systems	• How best to build capacity of the overall health system? • How to strengthen country leadership in understanding and implementing HSS? • Is it effective to invest in future leaders or is this something we cannot control? And, if effective, where should we be investing (e.g. diaspora, academics, politicians) to ensure there will be future leaders? • How best to work through and support local people, organisations and systems for research in insecure areas? • What methodological approaches build the capacity of local people to engage in research?
Actors & accountability	• Roles of various actors in states with weak governance • Accountability mechanisms for national and local government and international actors	• What role does the private sector play in providing health services in FCAS? And how can private provision be regulated to ensure that it promotes (rather than reduces) health equity? • What are the power relationships underlying different processes of accountability? (e.g. between donors or international NGOs and government, central government and local authority, different levels of the state and citizens) • How can international actors (UN, international NGOs, donors) be more effectively held accountable for their HSS activities? • What incentives help actors to be more accountable? And what are the consequences of the failure of accountability?
Community	• Community involvement and empowerment • Community readiness to participate in HSS • Roles of community-based providers	• What are the best approaches to bring community actors into full partnership with national health systems in order to strengthen the linkages between both systems: community system and health system? • What are the determinants of community readiness? And how can the level of community readiness best be increased in order for a community to participate in HSS? • What is the sustainability and quality of services provided by volunteer, versus paid, community health workers? • How to support community-based programming (CBP) beyond the conflict period? And what are successful and scalable models of CBP in post-conflict and fragile states?
Healthcare delivery	• Innovative approaches to service provision and best service delivery models • Quality of care and impact of quality improvement on HSS	• What healthcare delivery models work best in these contexts? Is this the ‘basic package of health services’ contracting model or any other model? And what kind of actors can best implement such models and deliver the best results? • How can quality and performance of healthcare providers best be measured in these contexts? • How can fragile states learn from stable low- and middle-income countries that have achieved improvements in quality of care in their health systems?
Health workforce	• Human resources for health management • Education and training of health workforce	• What kind of external support is most effective in supporting health managers in acute crisis? And how can you provide support that does not undermine the health workforce in these situations? • How best to build an appropriate health workforce post-conflict? • How can we move beyond the current in-service training focus and develop cadres of staff in conflict or crisis contexts rather than waiting for post-crisis situations?
Health financing	• Best finance practices in relation to aid and the political economy of aid • Results-based financing • Universal health coverage	• How much donor aid is enough or too much to instigate and maintain HSS while enabling country leadership? • How are funds channelled in FCAS? Are there any available successful models? • What are the specific opportunities and challenges of results-based financing in these contexts? • How does a vision for universal health coverage influence subsequent health system performance? • What funding schemes are being used? And are there any important mechanisms that are under-documented (e.g. Revolving Drug Funds or community financing)?

### Transition & sustainability

Research needs in this theme addressed the transition from humanitarian to development approaches, sustainability and rebuilding of a post-conflict health system. This relates to questions around the process of HSS and how best to do this in a transitional environment. Research questions on the transition from humanitarian aid into recovery support were also raised during the Evidence Aid prioritisation in June 2013 [16]. With the introduction of the sustainable development goals, sustainability has gained priority on the wider development agenda until 2030 [28].

### Resilience & fragility

The need for more research on resilience was particularly highlighted at the group session at the Health Systems Global Conference in September 2014. As the Ebola outbreak in West Africa was at its peak around that time, a link was made between this crisis and resilience. An increased interest in health system resilience and fragility due to the Ebola crisis is also reflected in the recent literature [3, 29]. Kruk et al. [3] propose a health systems resilience framework with definable characteristics that might be useful for future research in this area. Fragility, which some view as being on the opposite end of a spectrum to resilience [14], and its relationship with HSS was another research need that was raised. HSS has been described as state-building in the health sector [21], although, thus far, the relationship between health systems and state-building has been largely theoretical [17, 21, 30]. In order to achieve a better understanding of the relationship between fragility and HSS, there is a need for more empirical research on the link between state-building and the health system.

### Equity & gender

This study identified a need for more health systems research in the area of equity and gender. Ranson et al. [23] explored the topic of equity in conflict-affected states and concluded that more research is needed on how to effectively promote health equity in such states. Their study also raised the need for more research on the relationship between more inclusive health programming and conflict. Equity looks at avoidable and unjust differences in social groups in general, and one such social group that was specifically mentioned in this study were women. A recent narrative literature review concluded that there was limited literature on gender equity in health system reform in post-conflict settings [31] and not much clarity on “*what a gender equitable health system would look like*” (p. 12), which confirms our findings on the need for more research in this area.

### Accessibility

Participants identified the need for a better understanding of factors influencing access to health services. Physical, financial and conflict-related factors were mentioned, with the conflict-related factors short-listed. Referral systems and emergency care access was a related priority research area.

### Capacity building

Participants came up with many questions related to capacity building, with the question ‘How best to build capacity of the overall health system?’ seen as central. Additionally, this study highlighted a need for inquiry on capacity building of local researchers and information systems. Research capacity building is not just desirable in FCAS but also in other low-income countries [32]. While donors like DFID recognise this need [33], and there are some success stories [30, 34], more evidence is needed on best practices.

### Actors & accountability

More research on the roles of various actors in HSS and service provision is needed. A wide variety of actors were mentioned, including national governments, civil society, international NGOs, faith-based organisations, health partnerships, diaspora, and public and private sectors. More clarity on the roles of international NGOs and the private sector was found particularly pertinent in countries with weak governments. Besides greater clarity on the roles of various actors, there is also a need for a better understanding on how to hold these actors accountable, which has been raised before [17]. Future research may build upon work by the World Bank [35] that suggests international donors play an important part in the compact relationship.

### Community

Community was a research theme that was particularly discussed during the webinar. Research needs that were put forward included those on community involvement and empowerment and community readiness to participate in HSS. Additionally, the need for clarification on the role of community-based providers was raised. A previously published global systematic review on community health workers [36] might be consulted by those interested to further research on this topic.

### Healthcare delivery

A research theme that emerged was healthcare delivery, which is also one of the WHO health system building blocks [37]. The need for more research on innovative approaches to service delivery and best service delivery models was prioritised by participants. A commonly used health service delivery model in post-conflict settings is contracting non-state providers to deliver health services on behalf of the government. Previous research shows promising results in rapid expansion of services, but longer term effects have not been sufficiently researched [38, 39] and would therefore benefit from further investigation. In addition, this study found that future research should explore ways to improve the quality of service delivery in FCAS, possibly by learning from successful case studies in stable low- and middle-income countries. Types of healthcare found important for more exploration in FCAS include primary, maternal and mental healthcare.

### Health workforce

Another research theme that came out of this study, and also a health system building block, is the health workforce. Human resources for health (HRH) management and the education and training of health workers were short-listed research needs within this theme. These research needs also came up in a priority setting exercise into HRH in low- and middle-income countries [40], which suggests that these are not limited to FCAS. A literature review on HRH management in post-conflict health systems found that the limited research conducted thus far focused on the early post-conflict period and relied on secondary data, and advocated for more primary research on workforce supply, distribution and performance [41].

### Health financing

Another health system building block that was identified as a research theme is health financing. Within this theme, one of the research needs found pertinent by participants was related to aid, including best financing practices and their political economy. As strengthening health systems in FCAS is often highly dependent upon donor aid, this raises many economic, political and moral questions. There is a clear link here with capacity-building and accountability themes for the reason that aid has the potential to undermine national leadership and to interfere in the accountability relationship between a national government and its citizens [35]. Results-based financing was another research need short-listed in this study. The need for more research on payment and incentives systems was also raised in a review of the literature on health financing in fragile and post-conflict states [19]. Universal health coverage was an over-arching research need highlighted by study participants.

### Other research needs

Study participants also highlighted the need for specific types of research, including more policy analyses, implementation research, and innovative and inclusive research approaches. The need for better quality research was also highlighted as was the need for locally relevant research. The inclusion of local partners was a proposed solution by several participants because these generally have a better understanding of the socio-cultural and political environment. How best to include these local partners links to questions around research capacity-building. Further exploration of transferability and appropriateness of research and policies from one context to another was also prioritised in this study.

### Sub-group differences

Although the aim was to reach overall consensus on priority questions, it is interesting to note some differences which emerged between participants of different professional backgrounds. For example, comparing the numbers of times survey participants mentioned certain research needs, we note the following differences of emphasis:

- ° Academics and local implementers more often mentioned research needs related to capacity building (including health system, leadership, HRH and research capacity building);
- ° Funders and local implementers mentioned ‘actors’ more often;
- ° Local and international implementers mentioned ‘health financing’ more often than academics and slightly more often than funders;
- ° Local implementers were the only ones to mention ‘learning from stable settings’;
- ° Local and international implementers mentioned disease-related research needs (like maternal health, mental health) far more often (almost six- and four-fold, respectively) than academics and funders;
- ° Funders were more interested in ‘health information’; compared to academics, funders mentioned this research need 15 times more often than them and seven times more often than international implementers (the biggest group from our sample);
- ° Local implementers mentioned ‘leadership’ almost twice as often as academics and funders.

We also analysed difference by sex but these were less significant.

### Reflection on the consultative process

Our overall reflection on the process is that there may not be an ideal way of conducting priority-setting exercises – each approach and sequence has pros and cons. In our case, we were able to engage a diverse group of stakeholders at different points in the exercise, but (see limitations below) the topic and consultative techniques meant that the balance across stakeholder types was not always even. This will have influenced the final agenda (for example, the predominance of academics in the refinement stages may explain the absence of health information systems, which were more highlighted by funders, and leadership, which was a bigger concern to implementers). Similarly, while it was feasible to get lists of topic areas, it was harder to convert these into more specific research questions – to do this a final expert workshop stage had to be added (which had not originally been planned). The type of engagement permitted by, for example, webinars, does not allow the closer group-work which is needed to develop more detailed questions. Having a clear plan but being able to be pragmatic in how it is implemented may be essential to the success of such exercises, which often, as in this case, turn out to be more intensive than anticipated.

The original aim had been to develop a consensus around the key research areas in the field, but this is hard to develop when different participants are engaged in each of the stages of consultation. Further, many are firmly wedded to their areas of interest. Ultimately, the exercise may be more accurately termed a consultative agenda-setting process, in which a combination of wider engagement and expert honing combined to produce a set of topics which most stakeholders would recognise as important and valid, even if they are not exhaustive.

The decision was made early on not to seek a ranking of topics and this seems appropriate, in retrospect. The nature of the health system building blocks is that they are closely inter-dependent. Prioritising one over the other therefore makes little sense – each needs to be functional for others to work.

### Reflection on the research agenda

There is commonality between our research agenda and other published ones. Research agendas identified in health systems research priority exercises in low resource settings overlap – for example, in themes like health financing and human resources [42–44], equity [42, 43], community [42, 43], and accessibility [42]. There is also overlap, although of different themes, with exercises that focused on humanitarian settings, such as the themes of transition [16] and resilience [15], and on fragile and/or post-conflict states, such as the roles of actors (e.g. donors) [19] and incentives for health workers [20].

There are some areas which we might have expected to emerge more strongly, including on governance, health information systems (on local health needs and for accountability), and drugs and supplies. Several studies [11, 15, 40] highlight the importance of health information, not just as an important part of re-establishing functional health systems but also as an essential pre-requisite to health system research. Some are woven into the research needs that have been included (e.g. governance is related to the research need on accountability mechanisms for national and local governments under the ‘actors and accountability’ theme), but there were limited themes that emerged from the consultative process on the WHO building blocks [37], which may be a reflection of the type and interests of participants. This highlights the need to view this agenda as an important starting point, but by no means as exhaustive.

Some of the research needs in our agenda might be more of a priority for some FCAS than for others. Similarly, research questions presented in our agenda should be regarded as examples that need to be tailored to the specific context. The need for health systems research to have local relevance was highlighted by participants in this study as well as in previous studies. For example, authors of one study comment that “*HSPR* [health systems and policy research] *– unlike clinical or biomedical research – should be driven by understanding of local contexts*” [45]. That said, health systems research has been described as having a broad utility [4] and therefore could provide lessons learned for other similar contexts. However, in order to do so, the Task Force on Health Systems Research suggests that future research should better describe contextual factors and possibly include multiple countries [39]. An exploration of transferability and appropriateness, as highlighted by study participants, is, in light of this, important to broaden the utility of research across varying contexts.

### Limitations

This study has several limitations that should be noted when interpreting these findings. First, there was a lower than expected survey response rate. It is unclear why, although at the time of the survey the Ebola crisis in West Africa was at its peak, which could have made our target group less responsive to our survey request. Despite the sample size being smaller than anticipated (61 instead of 100), data seemed saturated as participants across the sample reported similar needs.

Second, although efforts were made to obtain a balanced sample in terms of demographic characteristics, more participants worked in international implementation (43 %) and the academic sector (31 %) than in local implementation (16 %) and funding (10 %). The perspectives of local implementers and funders are therefore likely underrepresented. In addition, researchers dominated the short-listing and refining stages of this exercise. A previous research priority exercise showed that researchers have different research agendas than policymakers [25], which is also suggested by our results on sub-group differences, and therefore this sampling issue likely influenced the overall research agenda.

Third, for feasibility reasons, our survey was only available in English and not in any other languages, which could have deterred some candidates from participating.

Despite its limitations, we do believe this consultative exercise achieved its goal of developing an initial research agenda on health systems in FCAS based on a systematic global consultation. We consulted a mix of male and female participants from across the world (survey participants were living in 28 different countries, of which 15 self-defined as FCAS), collectively bringing experience of health systems research in 56 different FCAS.

### Ways forward

The TWG on HS-FCAS aims to use this agenda to promote health systems research in these contexts. More specifically, this means assisting policymakers to commission research; persuading funders to support this research agenda; and encouraging researchers, particularly those in FCAS, to develop proposals for funding and, if needed, to develop the appropriate research capacity. The TWG is currently in discussion with one funder to support this area of research and have provided the agenda to help shape the call. We will maintain engagement with TWG members on a regular basis, e.g. at the 2-yearly Health Systems Research symposia, to ensure that the agenda remains contemporary and to encourage its use to guide research planning. This agenda-setting exercise itself contributed to the formation of a global community of policymakers, practitioners and researchers with an interest in health systems in FCAS. The consultative process supported the TWG HS-FCAS objective of expanding its membership and networks, which will help to take this research needs agenda forward.

## Conclusions

Fragility and conflict are on the increase and the relevance of understanding how to engage in strengthening and rebuilding health systems in these contexts is unlikely to diminish in the foreseeable future. Many organisations want to play a part, but the evidence base for guiding effective interventions in these complex environments is limited. There are real risks of unintended negative consequences of poorly designed and implemented interventions. More research will be needed, but funding to date is very limited. This makes establishing priority areas for health systems research topical and important.

This paper contributes to this arena by bringing together reflections on the process of consulting on the research agenda and presenting its results; both are important. Consultation itself gives higher priority to a topic and encourages participants to collaborate. The research agenda, while presented as a starting rather than end-point, also gives useful guidance on key areas for deepening knowledge. Without both higher profile and deeper focus, there is a real risk that FCAS areas will continue to fall behind in global health and development goals.

## Abbreviations

FCAS, fragile and conflict-affected states; HRH, human resources for health; HS, health systems; HSS, health systems strengthening; TWG, thematic working group

## Additional files

Additional file 1: Scoping review. This document shows the search strategy used for OVIDSP database searches, list of journals and organisations that were hand-searched via their websites, a flow diagram, and the list of records included in the review. (PDF 141 kb) Additional file 2: Online survey. This file shows the online survey, which was conducted as part of stage 2 (consultation on research needs) in this study. (PDF 590 kb)

## Footnotes

1 The Thematic Working Group on Health Systems in Fragile and Conflict-Affected States is a cooperation between the Health & Fragile States Network and the ReBUILD Consortium. As a working group of Health Systems Global, we aim to strengthen health systems research in fragile and conflict-affected states: http://healthsystemsglobal.org/twg-group/8/Health-Systems-in-Fragile-and-Conflict-Affected-States/.

2 The Health Systems in Fragile and Conflict-Affected States LinkedIn group brings together key actors on health in fragile and conflict-affected states and promotes research, policy and advocacy actions to develop and implement responsive and context-specific health systems: https://www.linkedin.com/groups/6611870.

3 The Health and Fragile States Network, established in 2007 and consisting of practitioners, researchers and funders, aims to contribute to the evidence base on health and fragile states; to influence policy and advocate collectively on a range of issues which impact on health in fragile states; and to support dialogue, debate and networking amongst those working in health and fragile states, and with those who work in other related sectors.

4 The ReBUILD Consortium conducts research for stronger health systems post conflict: www.rebuildconsortium.com.

5 This interactive map was developed via 'Google - Map Data' and can be accessed here: https://www.google.com/fusiontables/DataSource?docid=1nFPsjV6slJjg0v0uPaktlypYzPVc-5sYgDWHLpPD#map:id=3.

6 Third Global Symposium on Health Systems Research took place in Cape Town (2014), building on two previous symposia held in Montreux (2010) and Beijing (2012): http://healthsystemsresearch.org/hsr2014/home?qt-programme_at_a_glance=1.

7 In cooperation with the Thematic Working Group on Health Systems in Fragile and Conflict-Affected States, BioMed Central’s journal *Conflict and Health* published a series of articles entitled ‘Filling the void: Health systems in fragile and conflict affected states’: http://www.conflictandhealth.com/series/Filling_the_void.

8 The Thematic Working Group on Health Systems in Fragile and Conflict-Affected States organised a webinar on May 27, 2015, to present initial results on the agenda-setting exercise in order to invite discussion and comments. A recording of this 1-hour webinar can be accessed here: https://attendee.gotowebinar.com/recording/76320767420240641.
