# Supplementary material for: Health systems research in fragile and conflict-affected states: a research agenda-setting exercise
Source: Health Res Policy Syst. 2016 Jul 21;14:51. doi: 10.1186/s12961-016-0124-1 (PMC4955129; doi:10.1186/s12961-016-0124-1)
Supplement: Additional file 1: — Scoping review. This document shows the search strategy used for OVIDSP database searches, list of journals and organisations that were hand-searched via their websites, a flow diagram, and the list of records included in the review. (PDF 141 kb) [file 12961_2016_124_MOESM1_ESM.pdf]

Scoping Review

Search strategy used for OVIDSP database searches

Set	Search Statement
1.	((research or information) adj (agenda* or need* or priority or priorities or problem* or concern* or gap*)).mp.
2.	research priority setting/ or health priorit*/ or needs assessment.mp.
3.	1 or 2
4.	((health) adj (system* or service* or care or delivery or workforce or financing or information system or governance or leadership)).mp.
5.	exp Health/ or exp Health care/ or exp Health services/ or exp Health programs/ or exp Health policy/ or exp Health care costs/ or exp Health care workers/ or exp Health care utilization/ or exp Health centres/ or exp Health clinics
6.	4 or 5
7.	exp War/ or exp Emergency relief/ or exp Emergencies
8.	post-conflict/ or postconflict /or conflict-affected/or conflict affected /or conflict-ridden/ or protracted/ or wartorn/ or war*/ or fighting/ or insurgen*.mp.
9.	statebuilding/ or state-building/ or peace-building/ or peacebuilding/ or peacekeeping/ or health system strengthening/ or post-conflict reconstruction/ or postconflict reconstruction.mp.
10.	((fragile or unstable or fragility) adj (nation* or state* or country or countries or situation* or context* or setting* or region*)).mp.
11.	7 or 8 or 9 or 10
12.	3 and 6 and 11

List of journals and organisations that were hand-searched via their websites

Journals	Organisations
The Lancet & The Lancet Global Health	Alliance-HSPR
Conflict & Health	Health and Fragile States Network
Disasters	ReBUILD Consortium
Health Systems & Policy Research	WHO
Health Policy & Planning	WHO Global Health Workforce Alliance
Social Science & Medicine	OECD-INCAF
Medicine Conflict & Survival	Health Systems 20/20
Development in Practice	World Bank HIVE
European Journal of Public Health	McMaster Health System Evidence
PLoS Medicine	Google advanced search engine
Development Policy Review	ELRHA
International Journal for Equity in Health	
WHO Bulletin	
Global Health Governance	

Flow diagram

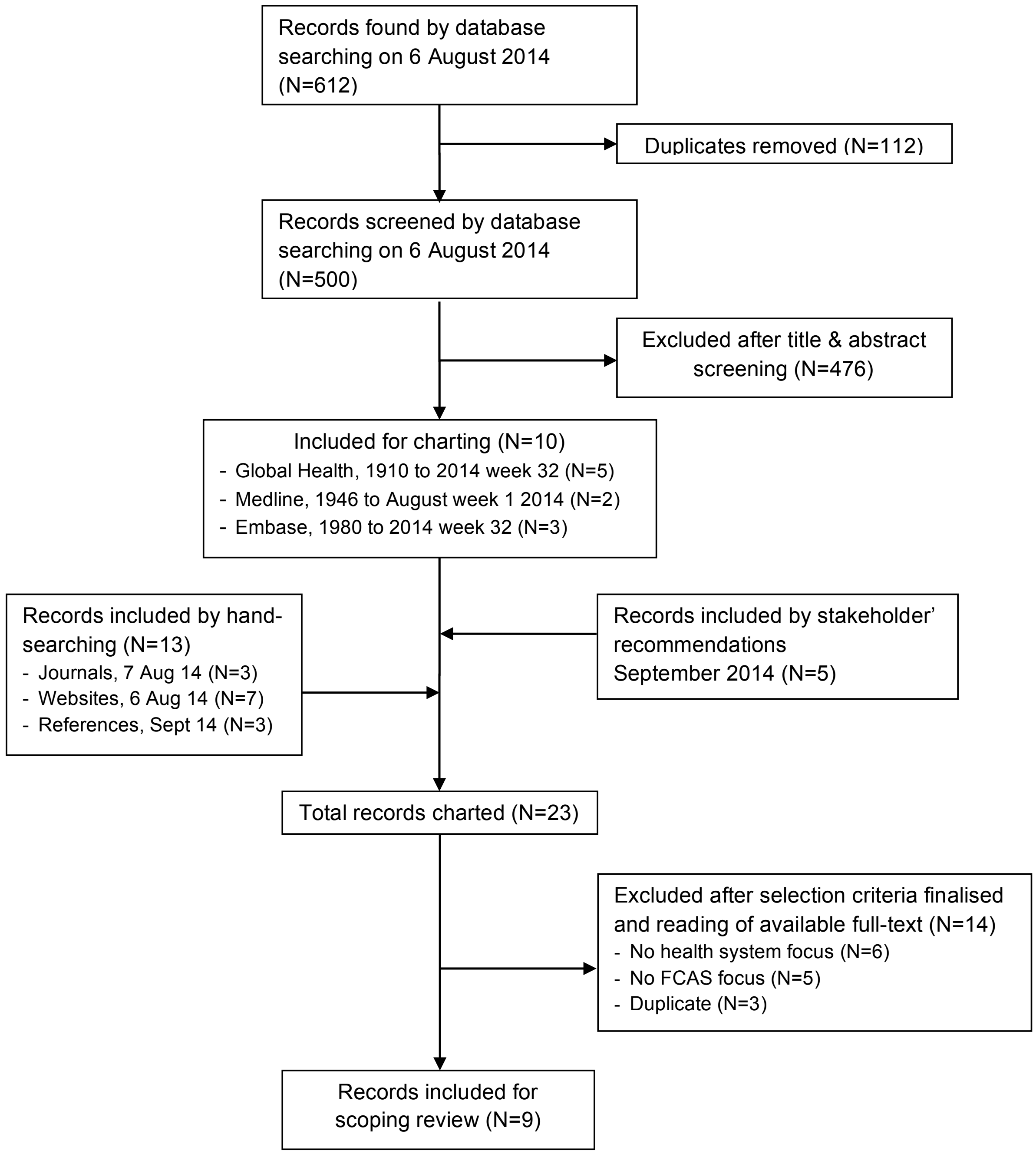

Records included in scoping review

	Lead Author	Year of publication	Title of article or report	Type of source
1	Kruk	2010	Rebuilding health systems to improve health and promote statebuilding in post-conflict countries: a theoretical framework and research agenda.	Journal article
2	Evidence Aid Priority Setting Group	2013	Prioritization of Themes and Research Questions for Health Outcomes in Natural Disasters, Humanitarian Crises or Other Major Healthcare Emergencies.	Journal article
3	Haar	2012	Health in fragile and post-conflict states: a review of current understanding and challenges ahead.	Journal article
4	Witter	2012	Health financing in fragile and post-conflict states: What do we know and what are the gaps?	Journal article
5	Tulloch	2011	HUMAN RESOURCES FOR HEALTH IN POST-CONFLICT SETTINGS	Report
6	Blanchet	2013	An evidence review of research on health interventions in humanitarian crises.	Report
7	Eldon	2008	Health Systems Reconstruction and State-building	Report
8	Alliance for Health Policy and Systems Research	2008	Neglected Health Systems Research: Health Policy and Systems Research in Conflict-Affected Fragile States	Report
9	Ranson	2007	Promoting health equity in conflict-affected fragile states. Prepared for the Health Systems Knowledge Network of the World Health Organisation's Commission on Social Determinants of Health	Report
