# Supplementary material for: Health systems research in fragile and conflict-affected states: a research agenda-setting exercise
Source: Health Res Policy Syst. 2016 Jul 21;14:51. doi: 10.1186/s12961-016-0124-1 (PMC4955129; doi:10.1186/s12961-016-0124-1)
Supplement: Additional file 2: — Online survey. This file shows the online survey, which was conducted as part of stage 2 (consultation on research needs) in this study. (PDF 590 kb) [file 12961_2016_124_MOESM2_ESM.pdf]

Health Systems in Fragile and Conflict Affected States

a subgroup of Health Systems Global

Health system research in fragile and conflict affected states

Welcome

Welcome to this survey that is being conducted part of a study on 'Health systems in fragile and conflict affected states: a global consensus-based research agenda setting exercise'.

Your decision to complete this survey is voluntary. We will maintain complete confidentiality and anonymity of your responses.

It should take you about 10-15 minutes to complete this survey, which consists of 4 sections:
1. Health system research in fragile and conflict affected states
2. Research needs
3. Your information
4. Comments & Next stage

Note that once you have clicked on the CONTINUE button at the bottom of each page you cannot return to review or amend that page

Continue >

Health system research in fragile and conflict affected states

Health Systems in Fragile and Conflict Affected States
a subgroup of Health Systems Global

Health system research in fragile and conflict affected states

Health system research in fragile and conflict affected states

There is no consensus in the research literature on what is meant by *fragile and conflict affected states* (and what countries are included) or *health systems research*. However for the purpose of this survey, this is what is commonly meant by these terms:
- *Fragile and conflict affected states* usually include countries that have been or currently are in conflict and/or have governments who lack the capacity or willingness to perform their core functions such as healthcare provision.
- *Health systems research* usually addresses different aspects of the health system (leadership/governance, health financing, health workforce, drugs/technologies, service delivery) in order to improve coverage, quality, access, safety and equity of health systems, and ultimately population health.

1. Based on your experience and familiarity with research literature, do you think that health system research in fragile and conflict-affected states is **different** from health systems research in other settings?

- Yes
- No
- Don't know

Why yes/no? *(Optional)*

2. Have you **ever been involved** in health systems research in fragile and/or conflict affected states?

- Yes
- No
- Don't know

If 'yes' did you experience **any challenges** in conducting your research?

- Yes
- No
- Don't know

i. If 'yes' what **kind of challenges** did you face?

Continue >

Survey testing only
Check Answers & Continue >

Health Systems in Fragile and Conflict Affected States
a subgroup of Health Systems Global

Health system research in fragile and conflict affected states

Research needs (open)

Research needs (open)

We are requesting you to **briefly outline what you think are the main research needs** on health systems in fragile and conflict-affected states. Please keep the following in mind when generating research needs:
- List needs that concern what you consider to be the top research need(s) to strengthen health systems in fragile and conflict-affected states.
- List at least 1 and up to a maximum of 5 research needs.
- Think of research needs that are relevant to the context of interest: fragile and conflict-affected states.
- Phrase needs that are based on your own experience and familiarity with research literature.
- Research needs do not have to be placed in order of priority.

3. Research need 1:

4. Research need 2: *(Optional)*

5. Research need 3: *(Optional)*

6. Research need 4: *(Optional)*

7. Research need 5: *(Optional)*

Continue >

Survey testing only

Check Answers & Continue >

Health Systems in Fragile and Conflict Affected States
a subgroup of Health Systems Global

Health system research in fragile and conflict affected states

Your information

Your information

8. Sex

○ Male ○ Female

9. In which country do you **currently** work (if more than one list the one you're mostly based)?

Select an answer

If you selected Other, please specify:

10. Do you have **any** experience of working in fragile and/or conflict affected states? If 'yes', please list at least 1 and up to a maximum of 5 countries (if you have experience of working in more than 5 countries list those you've got most experience with)

○ Yes
○ No

a. Country 1:
Select an answer

If you selected Other, please specify:

b. Country 2: *(Optional)*
Select an answer

If you selected Other, please specify:

c. Country 3: *(Optional)*
Select an answer

If you selected Other, please specify:

d. Country 4: *(Optional)*

Select an answer

If you selected Other, please specify:

e. Country 5: *(Optional)*

Select an answer

If you selected Other, please specify:

11. Would you say that **most** of your work takes place in an academic, implementing, or funding context?

- Academic (e.g. universities, research institutes)
- Local implementation (e.g. government, local NGO, policymakers)
- International implementation (e.g. international NGO)
- Funding (e.g. donors)
- Other *(please specify)*:

12. How did you hear about this survey?

- Email
- LinkedIn
- Other *(please specify)*:

Continue >

Survey testing only

Check Answers & Continue >

Health Systems in Fragile and Conflict Affected States
a subgroup of Health Systems Global

Health system research in fragile and conflict affected states

Comments & Next stage

Comments

13. Before you complete the survey, do you have any other comments to share with us, with respect to the research needs you stated? *(Optional)*

Next stage

In the next stage of this study we will be developing and creating consensus on the final research agenda. If you are interested in being involved in this next stage, please provide us with your email address.

14. Email address

Continue >

Survey testing only
Check Answers & Continue >

Health Systems in Fragile and Conflict Affected States
a subgroup of Health Systems Global

Health system research in fragile and conflict affected states

Thank you

Thank you for completing this survey as part of a study on 'Health systems in fragile and conflict affected states: a global consensus-based research agenda setting exercise'!

If you provided your email address, we will be in contact about how you can participate in developing and creating consensus on the final research agenda of this study in due time. If you didn't provide your contact details and would also like to be involved or if you have any other questions, please contact us via: aniek.woodward@lshtm.ac.uk

Please join our Health Systems in Fragile and Conflict Affected States **LinkedIn group** if you haven't done so already and stay in touch.
